# Association of ADP-Induced Whole-Blood Platelet Aggregation with Serum Low-Density Lipoprotein Cholesterol in Patients with Coronary Artery Disease When Receiving Maintenance Ticagrelor-Based Dual Antiplatelet Therapy

**DOI:** 10.3390/jcm12134530

**Published:** 2023-07-06

**Authors:** Bernadeta Chyrchel, Olga Kruszelnicka, Ewa Wieczorek-Surdacka, Andrzej Surdacki

**Affiliations:** 1 Second Department of Cardiology, Institute of Cardiology, Faculty of Medicine, Jagiellonian University Medical College, 2 Jakubowskiego Street, 30-688 Cracow, Poland; andrzej.surdacki@uj.edu.pl; 2 Department of Cardiology and Cardiovascular Interventions, University Hospital, 2 Jakubowskiego Street, 30-688 Cracow, Poland; 3 Department of Coronary Artery Disease and Heart Failure, Institute of Cardiology, Faculty of Medicine, Jagiellonian University Medical College, 80 Prądnicka Street, 31-202 Cracow, Poland; olga.kruszelnicka@gmail.com; 4 Center of Innovative Medical Education, Jagiellonian University Medical College, 7 Medyczna Street, 30-688 Cracow, Poland; esurdacka@gmail.com

**Keywords:** dual antiplatelet therapy, LDL cholesterol, platelet reactivity, P-selectin, ticagrelor

## Abstract

The degree of platelet inhibition in patients undergoing dual antiplatelet therapy (DAPT) affects cardiovascular outcomes after acute coronary syndromes (ACS) and/or percutaneous coronary intervention. Our aim was to search for correlates of residual ex vivo platelet reactivity and circulating soluble P-selectin (sP-selectin), an index of in vivo platelet activation, in patients being treated by DAPT with ticagrelor. Adenosine diphosphate (ADP)-induced platelet aggregability (by multiple electrode aggregometry) and plasma sP-selectin were estimated in 62 stable post-ACS subjects (46 men and 16 women; mean age: 64 ± 10 years; 30 with type 2 diabetes (T2DM)) undergoing maintenance DAPT with ticagrelor and aspirin. These patients did not exhibit heart failure or other relevant coexistent diseases except for properly controlled T2DM, mild renal insufficiency, and hypertension. We also assessed this in 64 subjects on clopidogrel-based DAPT matched for age, sex, and T2DM status. ADP-induced platelet aggregation was below the optimal levels (190–460 arbitrary units (AU) * min) in most patients receiving ticagrelor-based DAPT, especially in those with below-median (<1.9 mmol/L) serum concentrations of low-density lipoprotein cholesterol (LDL-c) (128 ± 61 vs. 167 ± 73 AU * min for below-median and above-median LDL-c, respectively, *p* = 0.025). In contrast, platelet reactivity did not differ by LDL-c on clopidogrel-based DAPT (246 ± 101 vs. 268 ± 108 AU * min for below-median and above-median LDL-c, respectively, *p* > 0.4). Plasma sP-selectin was found to be unrelated to serum LDL-c when receiving DAPT with ticagrelor (*p* > 0.4) or clopidogrel (*p* > 0.8). In conclusion, our preliminary observational study suggests the association of lower residual ex vivo platelet aggregability with better LDL-c control in patients undergoing ticagrelor-based maintenance DAPT, which does not appear to be reflected by plasma sP-selectin. Whether the serum LDL-c level should be considered among the factors affecting the degree of platelet inhibition for those treated with ticagrelor-based DAPT needs to be investigated in larger studies.

## 1. Introduction

Elevated residual platelet reactivity in patients undergoing dual antiplatelet therapy (DAPT) is a predictor of ischemic cardiovascular (CV) events in patients after acute coronary syndromes (ACS) and/or percutaneous coronary intervention (PCI) [1,2,3,4,5]. A meta-analysis by Aradi et al. [6] of the individual data of 20 839 patients on aspirin and a thienopyridine-type P2Y_12_ antagonist (97% on clopidogrel) supported the concept of a “therapeutic window” of P2Y_12_ receptor inhibition to minimize the risk of stent thrombosis and bleeding events. According to an updated expert consensus statement from 2019 [7], although platelet function testing may provide useful prognostic information after both ACS and elective PCI, the use of tailored DAPT is not routinely recommended. However, the individualized DAPT strategy may be considered an optional tool in specific clinical scenarios for DAPT escalation or de-escalation. 

Nevertheless, in contrast to observational and small randomized studies, no large-scale randomized trials have been able to demonstrate the clinical benefits of platelet function-guided DAPT escalation. This is in contrast to the promising results obtained in patients without high platelet reactivity on clopidogrel undergoing DAPT de-escalation from prasugrel to clopidogrel in the TROPICAL-ACS study [7,8]. Notably, it was suggested [9,10] that this phenomenon might be attributable to the continuous modulation of in vivo platelet aggregability by a three-way synergistic interaction between P2Y_12_ blockade, nitric oxide (NO), and prostacyclin (PGI_2_) [9,11,12,13]—a fact not reflected by the ex vivo assessment of platelet reactivity. Consequently, it was proposed that the combination of ex vivo platelet function testing and assessment of endothelial function or endothelial mediator production might improve the prediction of thrombotic events in individual patients [9,10].

The identification of clinical and biochemical correlates of both in vivo and ex vivo residual platelet reactivity in patients receiving DAPT with potent antagonists of a P2Y_12_ adenosine diphosphate (ADP) receptor is of clinical importance for optimizing the treatment of post-ACS subjects. Recently, we reported no association between ex vivo ADP-induced platelet aggregability and endothelial biomarkers in an observational study aimed at comparing biochemical indices of endothelial function in a relatively homogenous group of stable post-ACS patients undergoing DAPT with ticagrelor vs. clopidogrel [14]. 

Notably, multiple factors affect the reactivity of circulating platelets, in part by an interference with the NO-dependent and PGI_2_-mediated platelet inhibitory pathways [15]. The aim of the present analysis was to assess the previously described subjects undergoing ticagrelor-based DAPT for correlates of ex vivo platelet reactivity and circulating soluble P-selectin (sP-selectin), an index of in vivo platelet activation. These patients did not have any relevant coexistent diseases except for well-controlled type 2 diabetes (T2DM), mild renal insufficiency, or hypertension [14].

## 2. Materials and Methods

### 2.1. Patients

As described previously [14], the study design assumed similar numbers of patients aged 30–75 years with and without T2DM. In the design, the patients, recruited from 291 pre-screened consecutive hospitalized patients in a stable clinical condition on uneventful DAPT with ticagrelor or clopidogrel for 1–3 months after PCI for an ACS (non-ST-segment elevation myocardial infarction (NSTEMI), ST-segment elevation myocardial infarction (STEMI) and unstable angina in 42%, 33% and 25% of patients, respectively) were matched for age and sex, and met the pre-defined inclusion and exclusion criteria. 

The present analysis focused on 62 patients receiving DAPT with ticagrelor (mean age 64 ± 10 years; 46 men and 16 women), including 30 subjects with properly controlled T2DM. Additionally, in order to identify ticagrelor-specific associations, after finding the correlates of platelet reactivity on ticagrelor-based DAPT, we also estimated the relationship between these characteristics and platelet reactivity in 64 subjects on clopidogrel-based DAPT (mean age 65 ± 10 years; 46 men and 18 women) who were matched for age, sex, and T2DM status, as previously described [14]. Clopidogrel-based DAPT was administered in post-ACS subjects with contraindications to potent P2Y_12_ receptor antagonists, mostly at high bleeding risk.

At the time of blood sampling for platelet reactivity, the study subjects were receiving—in addition to low-dose aspirin and ticagrelor (90 mg b.d.) or clopidogrel (75 mg o.d.)—an angiotensin-converting enzyme inhibitor (ACEI) (or angiotensin receptor blocker (ARB)), a high-intensity statin and proton pump inhibitor (PPI). Additionally, none of the study patients were treated with other lipid-lowering drugs. We applied a wide set of exclusion criteria, including an unstable in-hospital course; significant left ventricular systolic dysfunction or overt heart failure; an estimated glomerular filtration rate (eGFR) below 45 mL/min per 1.73 m^2^ of body surface area by the Chronic Kidney Disease Epidemiology Collaboration (CKD-EPI) equation; and any other relevant coexistent diseases listed in detail earlier [14]. 

As previously described [14], the Bioethical Committee of our University approved the study protocol (approval numbers: KBET/277/B/2013 and 1072.6120.143.2019, issued on 28 November 2013 and 30 May 2019, respectively), and all participants provided informed consent.

### 2.2. Procedure

Blood was drawn from an antecubital vein via routine blood sampling to assess the platelet reactivity and sP-selectin levels. This was performed after an overnight fast, but before the administration of morning doses of drugs, including DAPT. 

The ADP-induced platelet aggregation was estimated by multiple electrode aggregometry (MEA) (Multiplate analyzer, Dynabyte, Münich, Germany) in a portion of thrombin inhibitor-anticoagulated whole-blood diluted to (1:2) with 0.9% sodium chloride [14,16,17]. The platelets’ reactivity to ADP was expressed in arbitrary units (AU) * min, representing the area under the curve, and corresponded to an increase in electrical impedance (due to the adhesion of platelets to the sensor wires). This was plotted against time during a 5-minute continuous recording after the addition of exogenous ADP in a final concentration of 6.4 µmol/L [14,16,17]. 

Blood for sP-selectin assays was collected in ethylenediaminetetraacetic acid-coated tubes. Plasma was separated upon centrifugation and stored at −80 °C until assayed. sP-selectin was measured by a commercially available ELISA (R&D Systems, Minneapolis, MN, USA) (lower detection limit: 0.05 ug/L; intra-assay and inter-assay coefficients of variation: 8.9% and 11.9%, respectively) [14].

Glycated hemoglobin (HbA1c) was measured by high-performance liquid chromatography (D-10 Hemoglobin Testing System, Bio-Rad, Hercules, CA, USA). Other routine serum biochemical assays were performed using an automated laboratory system (Cobas Pro, Roche Diagnostics GmbH, Mannheim, Germany). Total cholesterol, high-density lipoprotein cholesterol (HDL-c), and triglycerides (TG) were measured via enzymatic methods utilizing cholesterol esterase/cholesterol oxidase and lipase/glycerol kinase, respectively. The formed products were subsequently assayed by colorimetry. The concentration of low-density lipoprotein cholesterol (LDL-c) was calculated using the Friedewald formula. C-reactive protein (CRP) was measured via immunoturbidometry, and creatinine levels were determined by spectrophotometry based on the Jaffe reaction with alkaline picrate.

### 2.3. Statistical Analysis

Values are shown as means ± standard deviations (S.D.), medians (interquartile range), or numbers with percentages. Intergroup comparisons were performed using Student’s *t*-test after checking data normality (using the Kolmogorov–Smirnov test) and variance homogeneity (using Levene’s test). Bivariate correlations were assessed by Pearson’s correlation coefficients (r). Multiple linear regression was applied to estimate independent associations between variables; the variables with univariate *p*-values below 0.1 were entered into the regression equation as candidate covariates. A *p*-value below 0.05 was assumed to represent significance. The data were analyzed using Statistica (data analysis software system, version 13.3.704.0; TIBCO Software Inc. (2017), Palo Alto, CA, USA).

## 3. Results

The detailed characteristics of the study subjects undergoing DAPT with ticagrelor or clopidogrel, both with and without T2DM, were presented earlier [14].

**Figure 1:**
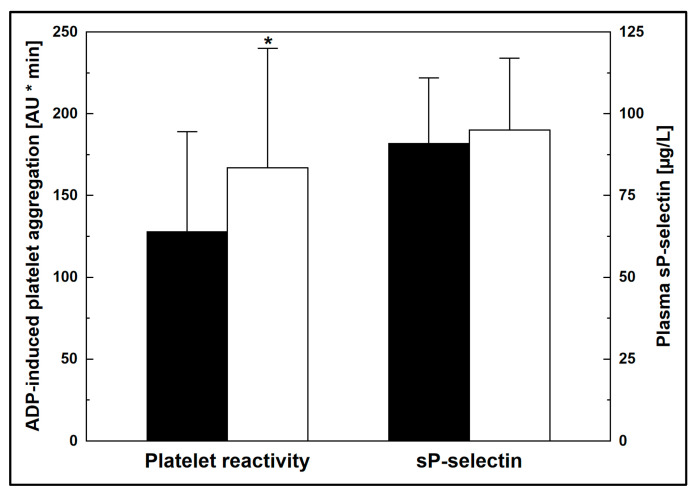
Ex vivo platelet reactivity and plasma soluble P-selectin (sP-selectin) in ticagrelor-treated patients with below-median (solid bars) and above-median (open bars) serum levels of LDL-c. Data are shown as means and S.D. * *p* = 0.025 vs. below-median LDL-c. ADP: adenosine diphosphate; AU: arbitrary units; LDL-c: low-density lipoprotein cholesterol.

In most ticagrelor-treated patients, the ADP-induced platelet aggregability was below optimal levels [14], defined as 190–460 AU * min by MEA [8]. Platelet reactivity correlated with serum LDL-c (r = 0.34, *p* = 0.008), and was lower in patients with below-median (<1.9 mmol/L) vs. above-median serum LDL-c (128 ± 61 vs. 167 ± 73 AU * min, *p* = 0.025) in the group undergoing DAPT with ticagrelor (Figure 1, Table 1). In contrast, platelet reactivity did not differ according to other metabolic parameters when stratified by the median values (Table 2). By multiple regression, higher LDL-c and older age were found to be the only independent significant predictors (*p* < 0.05) of increased ADP-induced platelet aggregation in ticagrelor-treated subjects.

Plasma sP-selectin was unrelated to any clinical parameters, except for its insignificant association with older age (r = 0.24, *p* = 0.09) in cases of ticagrelor-based DAPT. In particular, plasma sP-selectin was similar regardless of LDL-c concentrations (91 ± 20 vs. 95 ± 22 µg/L for below-median and over-median LDL-c, respectively, *p* > 0.4) (Table 1, Figure 1). The clinical and biochemical characteristics, as well as the medication used, were comparable in patients with below-median and above-median serum LDL-c (Table 1). 

**Figure 2:**
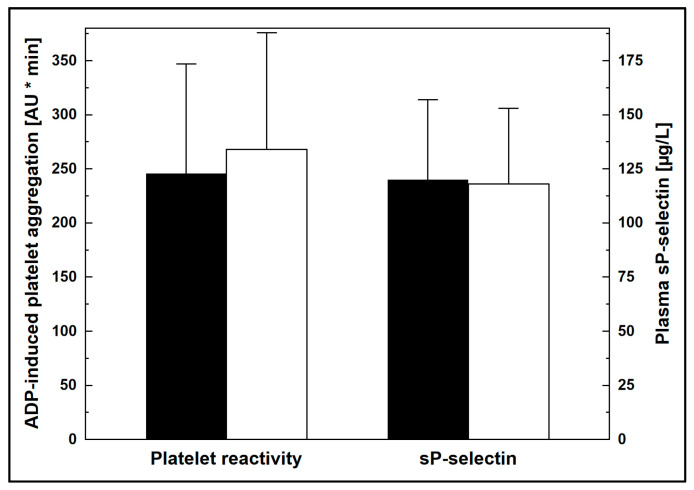
Ex vivo platelet reactivity and plasma soluble P-selectin (sP-selectin) in clopidogrel-treated patients with below-median (solid bars) and above-median (open bars) serum levels of LDL-cl. Data are shown as means and S.D. ADP: adenosine diphosphate; AU: arbitrary units; LDL-c: low-density lipoprotein cholesterol.

In clopidogrel-treated patients, serum LDL-c did not correlate with ADP-induced platelet aggregability (r = 0.13, *p* > 0.3). Platelet reactivity (246 ± 101 vs. 268 ± 108 AU * min, *p* > 0.4), plasma sP-selectin (120 ± 37 vs. 118 ± 35 µg/L, *p* > 0.8), and clinical and biochemical characteristics were similar in patients being treated with clopidogrel-based DAPT with below-median and above-median serum LDL-c, respectively (Figure 2, Supplementary Table S1). As previously shown [14], patients receiving DAPT with ticagrelor and clopidogrel and ticagrelor had similar characteristics, including serum LDL-c (1.9 ± 0.9 vs. 1.8 ± 0.8 mmol/L, respectively).

## 4. Discussion

Our salient finding was a lower residual ex vivo platelet reactivity on ticagrelor-based DAPT in patients with below-median serum levels of LDL-c, while circulating sP-selectin, an index of in vivo platelet activation, was similar regardless of the levels of LDL-c.

In a series of papers by the Novara Atherosclerosis Study Group, the investigators reported many correlates of elevated residual on-treatment platelet reactivity in patients undergoing DAPT with ticagrelor for 1–3 months, including advanced age [18,19], hypertension [19], T2DM (in particular with higher HbA1c) [20], higher neutrophil-to-lymphocyte ratio [21], lower Hb [22], decreased vitamin D [23], and increased homocysteine levels [24], whereas serum creatinine [25], uric acid [26], and BMI [27] were unrelated to platelet reactivity. 

On the other hand, in a meta-analysis of the patient-level data from 8 studies including 445 patients on a ticagrelor maintenance dose for at least 14 days, Alexopoulos et al. [28] identified current smoking status, older age, and BMI as univariate predictors of elevated platelet reactivity, and T2DM and advanced age as its only multivariate predictors. Nevertheless, in a subanalysis of 598 participants in the ISAR-REACT 5 trial, Ndrepepa et al. [29] recently reported a relationship between body size indices and platelet reactivity within 24 h of a patient taking a loading dose of prasugrel, but not ticagrelor. Moreover, T2DM status did not affect the platelet aggregability of 462 stable subjects on ticagrelor-based DAPT at 1 month after an ACS, in contrast to 315 subjects on prasugrel who exhibited higher platelet reactivity, especially in insulin-treated patients [30].

Notably, in neither one of those studies [18,19,20,21,22,23,24,25,26,27,28,29,30] correlated LDL-c or any other lipid parameter with increased platelet reactivity in patients treated with ticagrelor-based DAPT. However, higher LDL-c, in addition to HbA1c, was associated with increased platelet reactivity in mainly post-ACS subjects on prasugrel-based DAPT for 1–3 months [31], and higher pre-PCI LDL-c predicted future elevated platelet reactivity at 6 months of clopidogrel-based DAPT after elective PCI in univariate analysis, but not its multivariate form [32]. In addition, lower HDL-c, but not LDL-c, was associated with elevated platelet reactivity in patients receiving DAPT with clopidogrel, prasugrel, or ticagrelor at least 6 months after elective or ACS-related PCI [33].

The lack of correlation of sP-selectin levels and traditional risk factors, including LDL-c, in our post-ACS subjects is consistent with the results of most previous studies [34,35,36,37]. Admittedly, in a small study, Davi et al. [38] reported elevated sP-selectin and von Willebrand factor, a marker of endothelial dysfunction, in asymptomatic subjects with hypercholesterolemia and no evidence of CV disease. On the other hand, Blann et al. [35] described unchanged sP-selectin levels in patients with uncomplicated hypercholesterolemia, in contrast to increased sP-selectin in hypercholesterolemia with coexistent symptomatic CV disease. Conversely, von Willebrand factor concentrations were elevated irrespective of symptomatic status [35]. Furthermore, no associations of sP-selectin with traditional risk factors were reported in post-ACS subjects treated by DAPT with clopidogrel, despite an independent predictive value of sP-selectin with regard to the risk of future adverse CV events after ACS and/or PCI [36,37]. 

The partially inconsistent results of the previously mentioned studies, which were focused on determinants of platelet reactivity on ticagrelor-based DAPT, can hypothetically result from differences in patient characteristics, coexistent diseases, and concomitant medication. Additionally, different platelet function tests and different timings of blood sampling for platelet function testing with reference to both DAPT onset and maintenance dose administration could also be a factor. 

The fact that, in our study, neither T2DM nor HbA1c was associated with higher platelet reactivity (Supplementary Table S2) appears to be discordant with the results of some previous studies [20,39,40]. However, these conflicting results could be a consequence of the dependence of platelet reactivity on multiple factors, including glycemic variability [40] and short-term hyperglycemia [41], a fact not reflected by HbA1c. Moreover, possible effects of T2DM and glycemic control on platelet reactivity might be masked in the presence of a strong P2Y_12_ receptor blockade in our ticagrelor-treated patients.

Accordingly, the complex multifactorial nature of elevated platelet reactivity when undergoing DAPT limited the extrapolation of specific results to different clinical scenarios. Nevertheless, all reported determinants of high residual platelet reactivity on ticagrelor-based DAPT, including higher serum LDL-c in the present study, were established or putative CV risk factors [18,19,20,21,22,23,24,28,33].

As early as 1995, Notarbartolo et al. [42] reported associations between the lipid-lowering effect of simvastatin, simultaneous increases in threshold platelet-aggregating concentrations of ADP, and reduced urinary 11-dehydro-thromboxane B_2_ excretion in patients with type IIa hypercholesterolemia. Further studies identified mechanistic links between excessive platelet reactivity and LDL-c. 

First, the binding of oxidized lipids by the platelet CD36 receptor, a scavenger receptor implicated in atherogenesis and foam cell formation, is a recognized pathway linking dyslipidemia with excessive platelet activation [43,44]. Notably, CD36 ligation results in platelet activation via interference with the cyclic adenosine 3’,5’-monophosphate (cAMP)/protein kinase A (PKA) and cyclic guanosine 3’,5’-monophosphate (cGMP)/protein kinase G (PKG) signaling cascades, augmenting cAMP hydrolysis by phosphodiesterase 3A [45] and inhibiting cAMP-dependent PKA activation [45] and cGMP-dependent PKG activation downstream of cGMP synthesis [46]. 

Second, the release of NO by aggregating platelets [47] correlated negatively with mean arterial pressure and LDL-c, and decreased with the increasing number of traditional CV risk factors [48,49] in parallel to the degree of impairment of acetylcholine-induced forearm vasodilation [49]. This further strengthens the notion of depressed platelet-derived NO release as a contributor to excessive ex vivo platelet reactivity in patients with endothelial dysfunction, a common feature of most CV risk factors. 

Additionally, excessive oxidative stress, impaired NO bioavailability, and/or lower platelet responsiveness to NO, linked to the excessive accumulation of oxidized LDL, were also suggested [44,50,51,52] to explain the correlations of platelet reactivity with LDL-c and markers of endothelial activation in untreated patients with hypercholesterolemia [50]. Finally, in an experimental study, Nagy et al. [53] demonstrated the contribution of P2Y_12_ receptor pathway to agonist-induced platelet hyperreactivity in mice with mild hypercholesterolemia via thromboxane-dependent and thromboxane-independent (by protein kinase B [Akt] phosphorylation) pathways. 

Therefore, LDL-c has the potential to modulate platelet reactivity by multiple mechanisms, e.g., stimulation of the P2Y_12_/Akt and P2Y_12_/thromboxane pathways as well as interference with NO/cGMP/PKG and PGI_2_/cAMP/PKA signaling cascades, both upstream and downstream of the formation of cGMP and cAMP, the second messengers of NO and PGI_2_, respectively. 

The fact that, in the present study, we observed an association of elevated residual platelet reactivity, but not circulating sP-selectin, with higher LDL-c on ticagrelor-based DAPT appears to be attributable to a limited applicability of the results of ex vivo platelet function testing to in vivo conditions. Notably, a variety of in vivo modulators of platelet reactivity and their likely changes over time could have contributed to the lack of clinical benefits from tailored DAPT escalation guided by platelet function testing in all large-scale randomized clinical trials [7]. The fact that all of these major trials failed to meet their primary endpoints [7] may be due to the fact that patients with high ex vivo platelet reactivity might not have necessarily also exhibited excessive in vivo platelet activity. This could be linked to an in vivo three-way synergistic interaction between the P2Y_12_ blockade and continuously released endothelial mediators NO and PGI_2_ [9,11,12,13] that is not reflected by ex vivo assessment of platelet reactivity [9,10]. 

Importantly, the short-lived effects of NO, PGI_2_, and endogenous adenosine are likely to be missed during ex vivo platelet function testing in the whole blood due to the rapid degradation of adenosine [54] and hydrolysis of intracellular cyclic nucleotides by phosphodiesterases [54,55,56]. In particular, exogenous NO has been shown to produce only brief transient increases in platelet cGMP [55,56]. These peaked at 5–10 s [55,56] and returned to almost basal levels after 40–60 s, which mainly occurred due to the activity of phosphodiesterase type 5 [55]. Additionally, Nolte at al. [57] reported that most of platelet vasodilator-stimulated phosphoprotein (VASP)—a target of both cGMP/PKG- and cAMP/PKA-mediated phosphorylation—returned to its dephosphorylated isoform within 10 min of the removal of endothelial cells previously co-incubated with platelets.

Accordingly, it may be speculated that the stimulatory effects of LDL on platelet reactivity could be more easily revealed at low intraplatelet levels of cGMP and cAMP, as occurred during our ex vivo platelet function test. This is because the CD36-dependent effects, presumably attributable to oxidized LDL, occurred largely downstream of the synthesis of cGMP [46] and cAMP [45], being dependent on an impairment in the ability of cGMP and cAMP to activate PKG and PKA, respectively. In contrast, these effects may be hypothetically obscured in vivo by high and variable concentrations of cGMP and cAMP in circulating platelets exposed to NO, PGI_2_ [9,10,11,12,13] and adenosine [58], with the consequent lack of significant differences in sP-selectin between our patients with below-median and above-median LDL-c. 

Notably, the fact that Muller et al. [59] described a negative correlation of endothelial vasodilatory function (by peripheral arterial tonometry) with baseline sP-selectin levels (i.e., before clopidogrel administration) underscores the relevance of endothelial function to in vivo platelet activation. Moreover, the unique ability of ticagrelor to stimulate intraplatelet cAMP generation and NO release via enhanced accumulation of endogenous adenosine [58,60,61,62] could further potentiate the modulation of in vivo platelet function by endothelial mediators via a three-way synergistic interaction between the P2Y_12_ blockade, NO, and PGI_2_ [9,11,12,13]. These in vivo interactions—probably missed by ex vivo platelet function testing—might have masked NO- and PGI_2_-independent pathways linking serum LDL-c with platelet reactivity, thereby resulting in similar circulating sP-selectin concentrations irrespective of the lipid profile in our study patients on ticagrelor-based DAPT.

Second, the enhancement of platelet expression of P-selectin (stored in α-granules) in mice with mild hypercholesterolemia was weaker than the respective potentiation of the release of ATP (stored in dense granules) during ADP-induced platelet aggregation [53], which suggests a higher dependence of dense granule secretion than α-granule release on the hyperactivity of pathways downstream of P2Y_12_ receptors in response to elevated TC. Additionally, Barale et al. [50] described simultaneous decreases in LDL-c, ADP-induced platelet reactivity, and serum sP-selectin on simvastatin in hypercholesterolemic patients. However, pretreatment LDL-c correlated positively with ex vivo platelet reactivity, but not sP-selectin [50]. Therefore, the multifactorial modulation of circulating sP-selectin is likely to account for the lack of associations between plasma sP-selectin and LDL-c in the present study, which is in agreement with the vast majority of previous reports [34,35,36,37,50]. 

Therefore, the largely unpredictable impact of endothelial mediators, which was partially linked to pleiotropic effects of ticagrelor [63,64] and, likely, also statins [65,66] on in vivo platelet activation, augmented by a strong P2Y_12_ blockade [12,13] in addition to a relatively weak ability of mild hypercholesterolemia to stimulate platelet P-selectin expression [53] could jointly contribute to similar levels of sP-selectin regardless of LDL-c concentrations. This was despite the elevated ex vivo platelet reactivity in ticagrelor-treated patients with over-median LDL-c. 

Importantly, the majority of our ticagrelor-treated subjects hadlow ex vivo platelet reactivity, i.e., below a threshold value of 190 AU * min by MEA [8], which is consistent with the findings of a recent report [67]. This probably indicates an excessive risk of bleeding, especially in those with below-median LDL-c levels exhibiting the lowest ADP-induced aggregability in the present study, all the more because any hypothetical further inhibition of these platelets in vivo by endothelial mediators could also potentiate a bleeding risk. Notably, an increased risk of in-hospital major bleeding was reported in patients with recent ACS and low admission LDL-c in a large Chinese ACS registry [68]. This was compatible with the reduced bleeding risk at hypercholesterolemia reported in Western subjects treated by DAPT with prasugrel or clopidogrel [69,70]. It is noteworthy that an excess of hemorrhagic events was observed at an LDL-c concentration already below 2.3 mmol/L—comparable to a median of 1.9 mmol/L in the present study—in those ticagrelor-treated patients, while it was seen below 1.4 mmol/L in the subjects receiving clopidogrel [68]. 

Accordingly, it can be hypothesized that a stronger P2Y_12_ blockade by ticagrelor could facilitate the suppression of the previously described ability of hypercholesterolemia to enhance platelet reactivity via the modulation of signaling pathways downstream of P2Y_12_ receptors [53], meaning that excessive bleeding would appear at a higher serum LDL-c threshold for ticagrelor vs. clopidogrel [68]. The lack of association between platelet reactivity and LDL-c on clopidogrel-based DAPT in the present study is consistent with a lower respective threshold (1.4 mmol/L) in clopidogrel-treated subjects in the ACS registry [68], because LDL-c was above that threshold in most of our patients receiving DAPT with clopidogrel. 

Second, the previously described pleiotropic effects of ticagrelor [60,61,62,63,64] might predispose preferentially ticagrelor-treated subjects with low LDL-c to bleeding via the further inhibition of platelet reactivity [9,11,12,13] by enhanced accumulation of adenosine [58,60,61] and consequent NO release [62]. 

Regardless of the mechanisms involved, our preliminary results require further investigation. This is emphasized because the currently recommended LDL-c treatment goals in post-ACS subjects are even more strict, i.e., below 1.4 mmol/L, according to the European Society of Cardiology [71]. If confirmed, LDL-c may potentially be included among factors affecting the degree of platelet inhibition on ticagrelor-based DAPT and, possibly, also the individualized choice of DAPT intensity. Both guided and unguided DAPT de-escalation strategies in post-ACS subjects have recently been demonstrated to be associated with a tendency towards beneficial clinical effects [72,73,74], in contrast to major randomized trials focusing on DAPT escalation [7]. Additionally, the risk of intracerebral hemorrhage at low LDL-c levels is still debated [75], which also highlights the importance of tailored DAPT intensity in post-ACS patients.

Nevertheless, as our study was only observational, the hypotheses proposed above remain highly speculative and require validation in a large prospective study with platelet function testing and estimation of the risk of both ischemic and bleeding events. 

Some considerable study limitations should be acknowledged. First, the low number of patients and observational design constrain any far-going conclusions from our preliminary report. However, we analyzed a homogenous subgroup of the previously described ticagrelor-treated stable post-ACS subjects [14]. Second, we measured sP-selectin as a marker of in vivo platelet activation, although platelet P-selectin expression would be a better method for this purpose, because sP-selectin is released from both platelets and endothelial cells. Nevertheless, most circulating sP-selectin is of platelet origin [76]. Third, we estimated platelet reactivity by MEA, recently shown to be less sensitive and consistent than other platelet function tests in post-ACS patients undergoing DAPT [67]. Nonetheless, MEA was superior compared to the VASP-P assay for the prediction of future stent thrombosis in the PEGASUS-PCI study [77]. Fourth, a considerable diurnal variability of platelet reactivity was recently reported in ticagrelor-treated ACS subjects [78]. Nevertheless, platelet aggregatory responses were measured before the administration of the morning ticagrelor dose in all of our study patients. Fifth, the medication could potentially affect platelet aggregability and/or activation. However, all the study subjects received ACEI (or ARB) and high-intensity statin, which has previously been shown to decrease sP-selectin [79], and the proportion of subjects on other drugs was similar irrespective of LDL-c (Table 1, Supplementary Table S1). Finally, none of the diabetic study participants were on sodium-glucose co-transporter-2 inhibitors or glucagon-like peptide-1 receptor agonists, which are linked to beneficial CV effects.

## 5. Conclusions

Our preliminary observational study suggests the association of lower residual ex vivo platelet aggregability with better LDL-c control on ticagrelor-based maintenance DAPT, which does not appear to be reflected by circulating sP-selectin. This finding might hypothetically be linked to a probable modulation of in vivo platelet reactivity by short-lived endothelial mediators, with a possible contribution of the putative ability of ticagrelor to enhance endogenous adenosine accumulation. Whether serum LDL-c level might potentially be considered among factors affecting the degree of platelet inhibition on ticagrelor-based DAPT remains to be investigated in larger studies.

## Figures and Tables

**Table 1:** Platelet reactivity, plasma sP-selectin, clinical characteristics, and medications in patients receiving ticagrelor-based DAPT, stratified by LDL-c levels.

Variable	LDL-c		*p*
	Below-Median *n* = 31	Over-Median *n* = 31	
Platelet reactivity, AU * min	128 ± 61	167 ± 73	<0.05
sP-selectin, µg/L	91 ± 20	95 ± 22	n.s.
Clinical and biochemical characteristics			
Age, years	63 ± 10	65 ± 10	n.s.
Men/Women, *n* (%)	24/7 (77/23)	22/9 (71/29)	n.s.
T2DM, *n* (%)	14 (45)	16 (52)	n.s.
HbA1c ^a^, %	7.2 ± 1.0	7.4 ± 1.0	n.s.
BMI, kg/m^2^	30.0 ± 3.9	29.7 ± 3.6.	n.s.
HDL-c, mmol/L	1.0 ± 0.3	1.1 ± 0.3	n.s.
TG, mmol/L	1.5 ± 0.8	1.7 ± 0.9	n.s.
Arterial hypertension, *n* (%)	25 (81)	28 (90)	n.s.
Current smoking, *n* (%)	9 (29)	8 (26)	n.s.
LVEF, %	54 ± 10	49 ± 9	n.s.
Multivessel coronary artery disease, *n* (%)	19 (61)	23 (74)	n.s.
eGFR, mL/min per 1.73 m^2^	79 ± 19	77 ± 18	n.s.
CRP, mg/L	2.2 [1.3–3.5]	2.1 [1.2–3.2]	n.s.
Hb, g/dL	13.2 ± 1.9	13.0 ± 1.8	n.s.
Platelet count, 10^3^/µL	229 ± 71	238 ± 75	n.s.
Drugs beyond DAPT, ACEI/ARB, high-intensity statin and PPI			
β-blockers, *n* (%)	29 (94)	28 (90)	n.s.
Diuretics, *n* (%)	10 (32)	11 (35)	n.s.
Calcium channel blockers, *n* (%)	8 (26)	12 (39)	n.s.
Metformin, *n* (% of diabetic subjects)	13 (93)	15 (94)	n.s.
Sulfonyloureas, *n* (% of diabetic subjects)	3 (21)	4 (25)	n.s.
Insulin, *n* (% of diabetic subjects)	5 (36)	3 (19)	n.s.

Values are shown as mean ± S.D., median [interquartile range], or *n* (%). Abbreviations: ACEI: angiotensin-converting enzyme inhibitors; ARB: angiotensin receptor blockers; AU: arbitrary units; BMI: body mass index; CRP: C-reactive protein; DAPT: dual antiplatelet therapy; eGFR: estimated glomerular filtration rate (by the CKD-EPI formula); Hb: hemoglobin; HbA1c: glycated hemoglobin; HDL-c: high-density lipoprotein cholesterol; LDL-c: low-density lipoprotein cholesterol; LVEF: left ventricular ejection fraction; n.s.: not significant; PPI: proton pump inhibitor; sP-selectin: soluble P-selectin; T2DM: type 2 diabetes; TG: triglycerides. ^a^ only for diabetic subjects.

**Table 2:** Platelet reactivity in patients receiving ticagrelor-based DAPT, stratified by the median values of serum concentrations of HDL-c, TG and HbA1c, and median BMI.

	HDL-c, mmol/L		*p*	TG, mmol/L		*p*	HbA1c ^a^, %		*p*	BMI, kg/m^2^		*p*
	<1.0	>1.0		<1.6	>1.6		<7.3	>7.3		<29.9	>29.9	
Platelet reactivity, AU * min	152 ± 65	144 ± 60	n.s.	145 ± 63	150 ± 64	n.s.	147 ± 63	149 ± 64	n.s.	153 ± 66	143 ± 62	n.s.

Values are shown as mean ± S.D. Abbreviations as in Table 1. ^a^ only for diabetic subjects.

## Data Availability

The summary data supporting the main conclusions of this study are available upon request from the corresponding author.

## Supplementary Materials

The following supporting information can be downloaded at: https://www.mdpi.com/article/10.3390/jcm12134530/s1, Table S1: Platelet reactivity, plasma sP-selectin, clinical characteristics, and medication in patients receiving clopidogrel-based DAPT, stratified by LDL-c levels; Table S2: Prevalence of T2DM and the level of HbA1c stratified by platelet reactivity in the overall study patients receiving DAPT.
