# Supplementary material for: Association of ADP-Induced Whole-Blood Platelet Aggregation with Serum Low-Density Lipoprotein Cholesterol in Patients with Coronary Artery Disease When Receiving Maintenance Ticagrelor-Based Dual Antiplatelet Therapy
Source: J Clin Med. 2023 Jul 6;12(13):4530. doi: 10.3390/jcm12134530 (PMC10342583; doi:10.3390/jcm12134530)
Supplement: Supplementary file 1 [file jcm-12-04530-s001.zip › Supplementary Table S1.pdf]

Supplementary Table S1. Platelet reactivity, plasma sP-selectin, clinical characteristics and medication in patients receiving clopidogrel-based DAPT stratified by LDL-c levels.

**Variable**	**LDL-c**		***p***
	**Below-median** ***n*** **= 32**	**Over-median** ***n*** **= 32**	
Platelet reactivity, AU * min	246 ± 101	268 ± 108	n.s.
sP-selectin, μg/L	120 ± 37	118 ± 35	n.s.
Clinical and biochemical characteristics			
Age, years	65 ± 10	65 ± 9	n.s.
Men/Women, *n* (%)	25/6 (78/22)	21/10 (66/34)	n.s.
T2DM, *n* (%)	17 (53)	14 (44)	n.s.
HbA1c [a], %	7.3 ± 0.8	7.4 ± 0.9	n.s.
BMI, kg/m^2	28.8 ± 3.7	29.1 ± 3.8	n.s.
HDL-c, mmol/L	1.0 ± 0.3	1.0 ± 0.3	n.s.
TG, mmol/L	1.8 ± 0.8	1.6 ± 0.7	n.s.
Arterial hypertension, *n* (%)	27 (84)	29 (91)	n.s.
Current smoking, *n* (%)	10 (31)	8 (25)	n.s.
LVEF, %	49 ± 9	51 ± 10	n.s.
Multivessel coronary artery disease, *n* (%)	20 (63)	25 (78)	n.s.
eGFR, mL/min per 1.73 m^2	77 ± 18	76 ± 16	n.s.
CRP, mg/L	2.3 [1.2–3.7]	2.5 [1.4–4.0]	n.s.
Hb, g/dL	13.4 ± 2.0	13.3 ± 1.7	n.s.
Platelet count, 10^3/μL	221 ± 73	236 ± 79	n.s.
Drugs beyond DAPT, ACEI/ARB, high-intensity statin and PPI			
β-blockers, *n* (%)	28 (88)	30 (94)	n.s.
Diuretics, *n* (%)	11 (34)	9 (28)	n.s.
Calcium channel blockers, *n* (%)	11 (34)	12 (38)	n.s.
Metformin, *n* (% of diabetic subjects)	15 (88)	13 (93)	n.s.
Sulfonyloureas, *n* (% of diabetic subjects)	5 (29)	2 (14)	n.s.
Insulin, *n* (% of diabetic subjects)	6 (35)	4 (29)	n.s.

Values are shown as mean ± S.D., median [interquartile range] or *n* (%). Abbreviations as in Table 1.

[a] only for diabetic subjects
