# Supplementary material for: Association of ADP-Induced Whole-Blood Platelet Aggregation with Serum Low-Density Lipoprotein Cholesterol in Patients with Coronary Artery Disease When Receiving Maintenance Ticagrelor-Based Dual Antiplatelet Therapy
Source: J Clin Med. 2023 Jul 6;12(13):4530. doi: 10.3390/jcm12134530 (PMC10342583; doi:10.3390/jcm12134530)
Supplement: Supplementary file 1 [file jcm-12-04530-s001.zip › Supplementary Table S2.pdf]

Supplementary Table S2. Prevalence of T2DM and the level of HbA1c stratified by platelet reactivity in the overall study patients receiving DAPT.

	**ADP-induced platelet aggregability, AU * min**		
	**< 190**<br>***n* = 62**	**≥ 190**<br>***n* = 64**	***p***
T2DM, *n* (%)	28 (45)	33 (52)	n.s.
HbA1c [a], %	7.3 ± 0.8	7.3 ± 0.9	n.s.

Values are shown as mean ± S.D. or *n* (%). Abbreviations as in Table 1.

[a] only for diabetic subjects
